# Desmoplastic small round cell tumor of the abdomen: A case report

**DOI:** 10.1097/MD.0000000000035965

**Published:** 2023-11-10

**Authors:** Runze Li, Weizhen Liu, Lin Ye

**Affiliations:** a Department of Gastrointestinal Surgery, Union Hospital, Tongji Medical College, Huazhong University of Science and Technology, Wuhan, China.

**Keywords:** abdominopelvic tumor, case report, desmoplastic small round cell tumor, young male

## Abstract

**Rationale:**

Desmoplastic small round cell tumor (DSRCT) is a rare malignant tumor with poor prognosis, usually involving the peritoneum. There are currently no standardized treatment approaches. This study helped to further advance our understanding of DSRCT, and help to guide therapy.

**Patient concerns:**

The patient, a 19-year-old male, presented with left-sided back pain with no obvious cause and occasional abdominal pain, and underwent abdominal electron computed tomography examination in our hospital suggesting consideration of small bowel mesenchymal tumor with possible multiple implantation metastasis in the abdominopelvic cavity.

**Diagnoses:**

After surgical treatment, the pathology report suggested a DSRCT, and immunohistochemistry and fluorescence in situ hybridization revealed *EWSR1-WT1* gene rearrangement. Lung computer tomography and abdominal magnetic resonance imaging performed half a month later showed multiple solid nodules on the proximal septal surface of the right lung base, right posterior cardiac/right anterior inferior vena cava nodules, and multiple nodules in the abdominopelvic cavity, omenta, peritoneum, and around the liver or liver, all of which were considered as metastatic foci.

**Interventions and outcomes:**

Patient received 5 cycles of chemotherapy after surgery. The review results showed a smaller size than before. Currently, he continues to receive treatment.

**Lessons:**

The reported case has raised awareness of the importance of DSRCT in the treatment of chemotherapy, including its role in the differential diagnosis of abdominal tumors.

## 1. Introduction

Desmoplastic small round cell tumor (DSRCT) is a rare and aggressive mesenchymal malignancy with characteristic chromosomal translocation features.^[1]^ The first description of DSRCT was proposed by Gerard and Rosai et al in 1989, classified it according to the expression of different markers. It was not until 1991 that DSRCT was recognized as a separate pathological type.^[2]^ DSRCT is primarily a cancer of young adults, occurring at a median age of 20 years. The most common symptom of DSRCT is abdominal pain,^[3]^ and metastases are found at the time of diagnosis.^[4]^ DSRCT is currently diagnosed mainly based on histopathological, immunohistochemical, and cytogenetic studies to confirm characteristic chromosomal translocations.^[5]^

We present a recent case of abdominal DSRCT. The purpose of this study was to describe the microscopic pattern and cytological criteria of DSRCT and to describe our experience in the diagnosis and treatment of DSRCT.

## 2. Case presentation

**Figure 1.**
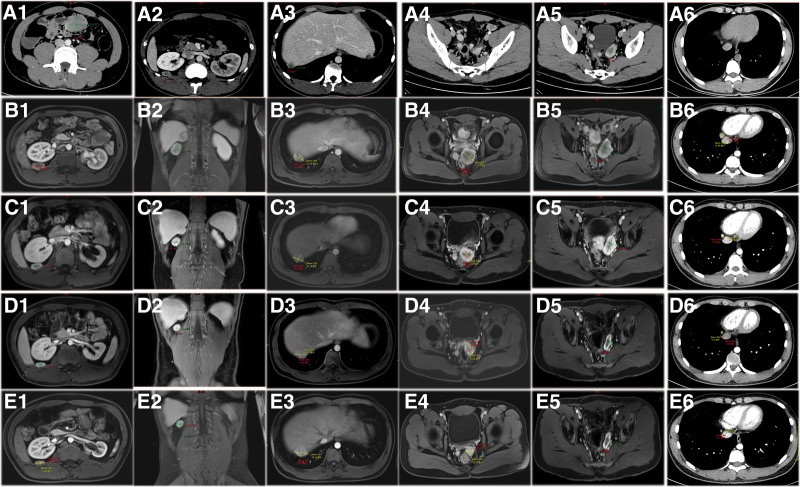
Preoperative and postoperative imaging diagnosis. (A) The small intestine area in the middle and lower abdomen could have a size of about 50 mm × 34 mm mass with heterogeneously enhancing (A1), and with multiple implantation metastasis in posterior peritoneum (A2), liver (A3), pelvic cavity (A4–5), and in right rear of heart/right front of inferior vena cava (A6). A follow-up examination 1 month after the surgery revealed that tumors were larger than those before the surgery in posterior peritoneum (B1–2), liver (B3), pelvic cavity (B4–5), and in right rear of heart/right front of Inferior vena cava (B6). Examination after receiving fifth chemotherapy showed that tumors was gradually reducing in posterior peritoneum (C1–2), liver (C3), pelvic cavity (C4–5), and in right rear of heart/right front of inferior vena cava (C6). Examination after receiving sixth chemotherapy showed that tumors were gradually reducing in posterior peritoneum (D1–2), liver (D3), pelvic cavity (D4–5), and in right rear of heart/right front of inferior vena cava (D6). A follow-up examination 7 month after the surgery revealed that tumors were gradually reducing in posterior peritoneum (E1–2), liver (E3), pelvic cavity (E4–5), and in right rear of heart/right front of inferior vena cava (E6).

A 19-year-old man presented with an abdominal mass. On October 19, 2022, contrast-enhanced computer tomography from the middle abdomen to the pelvic cavity was performed, and the results showed that the small intestine area in the middle and lower abdomen could have a size of about 50 mm × 34 mm mass with heterogeneously enhancing, and with multiple implantation metastasis in abdominal and pelvic cavity (Fig. 1A2–5) and implantation metastasis in right rear of heart/right front of inferior vena cava (Fig. 1A6) are considered for diagnosis.

**Figure 2.**
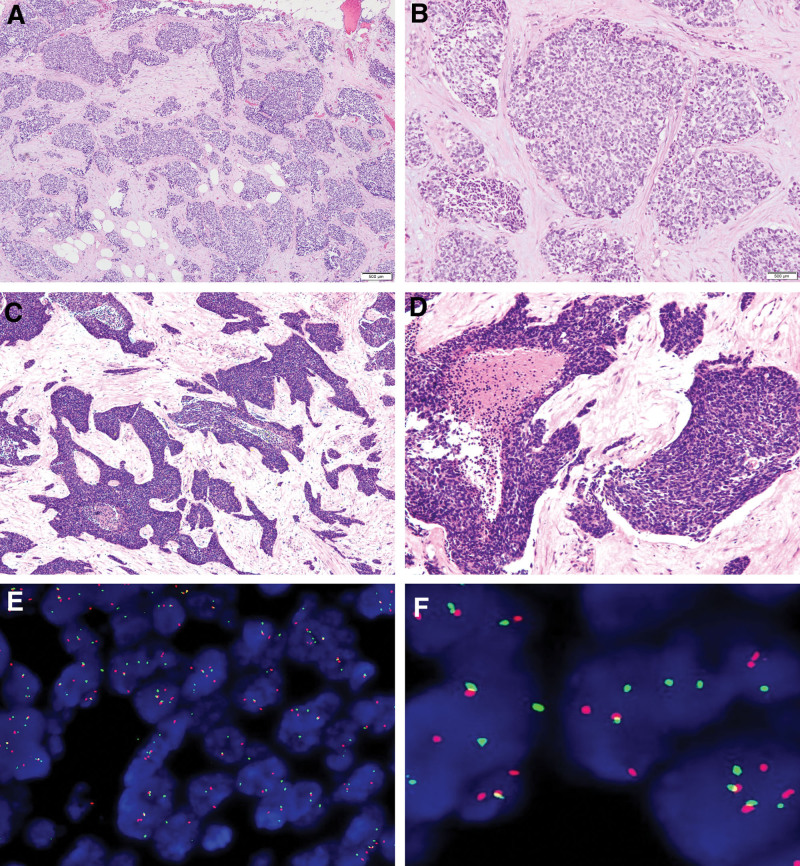
Histological findings of desmoplastic small round cell tumor. Intraoperative frozen section examination (A. HE 10×; B. HE 20×) and postoperative permanent pathological examination (C. HE 10×; D. HE 20×). Dual color break apart specific locus FISH probe targeting *EWSR1* gene at 22q12.2 chromosomal region; green and red signals mark the 5′ and 3′ ends of the gene respectively. (E) 200×; F 400×.

On October 24, 2022, he received abdominal tumor resection in our hospital. During the operation, we observed that harder tumors existed in the root of the mesentery of ileocecal and terminal ileum with invading the abdominal wall, and in the area between sigmoid colon and mesenteries of lateral margins, and in proximal jejunum near ligament of Treitz. Harder tumors in greater omentum abundantly disseminated. Postoperative pathology showed (abdominal tumor) DSRCT (Fig. 2A–D). Tumor cells expressed an epithelial marker, such as PCK and EMA. Desmin, NSE, WT1, TLE1, SATB2, and CD99 proteins were strongly expressed in the tumor cells. The positive rate of Ki-67 was about 70%. However, S-100, SALL-4, CD34, CD117, Syn, CgA, MyoD1, myogenin, ETV4, BCOR, NUT, SS18-SSX were negative. Fluorescence in situ hybridization revealed *EWSR1-WT1* gene rearrangement at 22q12, confirming the diagnosis of a DSRCT (Fig. 2E and F).

A follow-up examination 1 month after the surgery revealed that the chest, abdomen, and pelvic masses were significantly larger than those before the surgery (Fig. 1B1–6). The patient began receiving their first chemotherapy on December 26, 2022, with alternating treatment of VDC (Doxorubicin + Cyclophosphamide + Vincristine) and IE (Ifosfamide + Etoposide). The patient’s follow-up examination on January 27, 2023 showed no significant increase in chest, abdominal, and pelvic masses compared to before (Fig. 1C1–6). On March 22, 2023, patient had a measurable disease at baseline by response evaluation criteria in solid tumors and he was PR+ (Fig. 1D1–6). The patient’s follow-up examination on May 22, 2023 showed that the chest, abdomen, and pelvic masses were significantly smaller than before (Fig. 1E1–6). The patient plans to undergo the 9th chemotherapy on June 7, 2023.

## 3. Discussion

Pathologists named it DSRCT after discovering that the undifferentiated small round cell clusters in tumor tissue is surrounded by abundant desmoplasia. DSRCT usually occurs between the ages of 5 and 50 years, with an average age of 22 years. Nearly 85% to 90% of patients are male, but the proportion of women is slightly higher among patients younger than 20 years old at the time of diagnosis.^[6]^

In clinical practice, the signs and symptoms of DSRCT do not have specific manifestations, and most patients only present as abdominal masses. It is occasionally accompanied by pain, abdominal distention and/or ascites, constipation, weight loss, or other symptoms secondary to external mass effects (bowel obstruction) or damage to abdominal and pelvic organs.

This type of cancer is believed to originate on the surface of the peritoneum and has almost spread widely when discovered. Common metastatic sites include the liver, spleen, and lymph nodes above the diaphragm,^[7]^ but the disease may also occur in other areas, including the testes and central nervous system.^[8]^ With the increasing number of medical record reports, clinical physicians have come up with many different staging methods for DSRCT. Recent methods use imaging features to define moderate (no liver involvement or ascites), high-risk (liver involvement or ascites), and extremely high-risk diseases (liver involvement and ascites).^[3]^

In the majority of DSRCT cases, expression of Desmin, CK, EMA, and vimentin is positive. When both Desmin and CK are positive, it is considered an immunodiagnostic indicator specific for DSRCT. Vimentin positivity suggests that the tumor is derived from muscle fibroblasts. Such tumors can express epithelial, mesenchymal, neuroendocrine, and other immune phenotypes, and they are also cytogenetically specific.^[9]^ Thus, to a large extent, the direction of DSRCT cell differentiation is uncertain. In addition, the cells can produce *EWS-WT1* fusion genes.^[10]^

The present study had some limitations. First, termination of treatment is forced due to the epidemic in 2022, which might impact the effect of chemotherapy for DSRCT. Second, patient reviewed by magnetic resonance imaging from second time to now, while checked by computer tomography in first time, which might cause that clinical and follow-up information was not adequately compared.

Surgical resection, chemotherapy, and radiation therapy are currently the main treatment methods. In addition, according to the patient’s condition, interventional therapy, neoadjuvant chemotherapy, and neoadjuvant radiotherapy can be given. Recent studies have shown that complete surgical removal of tumors, including 1- to 2-mm-long implanted tumor bodies, can effectively reduce the recurrence rate of the disease. There are research reports that using intraperitoneal hyperthermia chemotherapy containing cisplatin can achieve long-term disease control.^[11]^ Despite the existence of multimodal treatment, the overall survival rate of DSRCT patients at 5 years is very low, ranging from 15% to 30%.^[12,13]^ The case in this report underwent 9 cycles of chemotherapy after surgery, and radiation therapy may be considered in the future.

## Author contributions

**Data curation:** Weizhen Liu.

**Supervision:** Lin Ye.

**Writing – original draft:** Runze Li.
